# Hyperbaric Oxygen Therapy in Modern Surgical Practice: Mechanistic Basis and Clinical Applications Across Specialties

**DOI:** 10.7759/cureus.102116

**Published:** 2026-01-22

**Authors:** José Emiliano González Flores, Daniela B Vázquez Hernández, Aarón Gonzalez Espinosa, Alfonso Sandoval Polito, Andrea Navalón Calzada, Edson O Romero Cázares

**Affiliations:** 1 Medical Education and Simulation, Tecnológico de Monterrey Campus Ciudad de Mexico, Mexico City, MEX; 2 Pediatric Anesthesiology, National Institute of Pediatrics, Mexico City, MEX; 3 Plastic, Aesthetic and Reconstructive Surgery, General Hospital of Mexico "Dr. Eduardo Liceaga", Mexico City, MEX; 4 Surgery, Tecnológico de Monterrey Campus Ciudad de Mexico, Mexico City, MEX; 5 Surgery, Hospital Español, Mexico City, MEX; 6 Medical Education and Simulation, Mexican Army, Secretariat of National Defense, Mexico City, MEX

**Keywords:** angiogenesis, chronic limb-threatening ischemia (clti), diabetic foot ulcer (dfu), flaps and grafts, hyperbaric oxygen therapy (hbot), perioperative care, radiation injury, reconstructive surgery, surgical wound healing, venous leg ulcers

## Abstract

Post-surgical wound complications remain a major driver of morbidity and cost, largely mediated by tissue hypoxia, dysregulated inflammation, and infection. Hyperbaric oxygen therapy (HBOT) increases dissolved plasma oxygen and creates a transient hyperoxic milieu that supports fibroblast activity, collagen cross-linking, angiogenesis, and host antimicrobial defenses. We performed a targeted narrative review of PubMed/MEDLINE (January 2020-November 2025); 89 articles were identified, and 38 met the inclusion criteria. Mechanistic evidence shows HBOT modulates redox signaling, downregulates pro-inflammatory pathways, optimizes hypoxia-inducible factor-1α (HIF-1α)-vascular endothelial growth factor (VEGF) dynamics, and balances matrix metalloproteinase/tissue inhibitor of metalloproteinase (MMP/TIMP) activity, thereby improving matrix quality and microvascular integrity. Clinically, across diabetic and vascular indications (diabetic foot ulcer, chronic limb-threatening ischemia, and venous/ischemic ulcers), adjunctive HBOT trends toward higher healing rates, improved limb preservation, and better graft take when added to standard care. In reconstructive and plastic surgery, HBOT supports threatened flaps and grafts and benefits burn management; aesthetic procedures show fewer complications and faster recovery. In trauma and orthopedic settings, early HBOT mitigates ischemia-reperfusion injury and aids soft-tissue salvage, while in oncologic/radiation contexts, it improves outcomes in late radiation tissue injury and irradiated reconstructive fields. Safety is favorable when typical regimens are used (2.0-2.5 ATA for 60-90 minutes, 20-40 sessions), with mostly mild, reversible adverse events. Evidence interpretation is limited by heterogeneity in protocols, endpoints, and patient selection. Future priorities include multicenter randomized trials, protocol standardization, biomarker-guided patient selection, and cost-effectiveness analyses. Overall, HBOT is a physiologically sound, clinically versatile perioperative adjunct that, when integrated into multidisciplinary pathways, can enhance healing and reduce complications across surgical specialties.

## Introduction and background

Background

Post-surgical wound complications remain a significant burden in contemporary surgery, accounting for considerable morbidity, prolonged hospital stays, and increased healthcare costs. Surgical wound dehiscence, infection, and flap or graft failure are most frequently associated with tissue hypoxia and impaired microvascular perfusion, two major barriers to adequate healing [1,2]. Although advances in surgical technique and perioperative care have improved outcomes, impaired wound healing persists as a major determinant of surgical success and patient recovery.

Hyperbaric oxygen therapy (HBOT) has emerged as a valuable adjunct in the management of complex and hypoxic wounds. By exposing patients to 100% oxygen under pressures exceeding one atmosphere absolute (ATA), HBOT markedly increases the amount of oxygen dissolved in plasma, thereby elevating tissue oxygen tension to levels that support reparative cellular processes. This hyperoxic environment enhances fibroblast proliferation, collagen synthesis, angiogenesis, and bacterial clearance while reducing tissue edema and inflammation [3,4]. These mechanisms together accelerate wound closure, strengthen tensile integrity, and reduce the risk of ischemic necrosis, critical factors for post-surgical recovery.

Rationale

Despite its well-established physiological foundation, the clinical integration of HBOT into surgical protocols remains inconsistent. Variability in pressure settings, session duration, and timing relative to surgery has led to heterogeneous outcomes among published studies. Nonetheless, a growing body of evidence supports its efficacy across a spectrum of surgical contexts.

Systematic reviews and randomized controlled trials have demonstrated that HBOT significantly increases healing rates and reduces major and minor amputations in patients with chronic and ischemic wounds [5,6]. In reconstructive surgery, it improves flap and graft survival, promotes neovascularization, and minimizes postoperative complications. Recent prospective data confirmed that HBOT combined with standard wound care led to faster epithelialization, enhanced granulation tissue formation, and lower minor amputation rates compared to standard care alone [6].

While encouraging, these findings also reveal gaps in standardization and patient selection. The optimal timing, dosage, and duration of HBOT for surgical indications remain undefined. Consequently, a critical synthesis of the available evidence is required to delineate the role of HBOT in perioperative wound management and to guide future research and clinical implementation [7].

Objective

This comprehensive narrative review aims to consolidate current physiological and clinical evidence regarding the role of HBOT in post-surgical wound healing. Specifically, it seeks to elucidate the cellular and molecular mechanisms through which HBOT promotes tissue repair and angiogenesis, to summarize the clinical efficacy of HBOT across various surgical specialties, including reconstructive, vascular, orthopedic, and oncologic surgery, and to identify the current limitations, controversies, and research priorities necessary to support the development of standardized perioperative HBOT protocols.

## Review

Methods

A targeted literature search was conducted using the PubMed/MEDLINE database to identify studies evaluating the role of HBOT in surgical and reconstructive wound healing. PubMed was chosen for its comprehensive coverage of peer-reviewed biomedical literature and for indexing the principal journals in surgery, hyperbaric medicine, and wound research. The search was limited to publications from the past five years (January 2020-November 2025) to ensure the inclusion of the most recent clinical evidence and to reflect current treatment protocols. The last search was conducted on November 12, 2025.

The search strategy combined both Medical Subject Headings (MeSH) and free-text terms using Boolean operators. The following search string was applied: ("hyperbaric oxygen therapy" OR "HBOT") AND ("surgical wound" OR "reconstructive surgery" OR "flap" OR "graft" OR "burn" OR "ischemic ulcer" OR "radiation injury").

This five-year window was selected to capture contemporary data on perioperative HBOT use, as older studies often relied on outdated pressure regimens and non-standardized protocols. The search yielded a total of 89 articles, of which 38 met the inclusion criteria after screening for relevance and full-text availability. All 38 included studies were incorporated throughout the manuscript to support both the introductory context and the narrative synthesis, consistent with the methodological flexibility of a structured narrative review.

Inclusion criteria consisted of the following: (1) peer-reviewed articles published between 2020 and 2025; (2) clinical studies, systematic reviews, or meta-analyses evaluating HBOT in surgical, reconstructive, or ischemic wound contexts; (3) studies reporting quantitative or descriptive outcomes such as wound closure, angiogenesis, flap or graft survival, or complication rates; and (4) publications in English with accessible full text.

Exclusion criteria included the following: (1) animal or in vitro studies without translational correlation; (2) conference abstracts, editorials, or narrative commentaries lacking primary data; and (3) studies investigating HBOT exclusively for non-surgical indications such as carbon monoxide poisoning or decompression sickness.

The selection process was performed manually. Titles and abstracts were screened for relevance, and full texts of potentially eligible studies were reviewed. Data extraction included study design, patient population, HBOT parameters (pressure, duration, and number of sessions), outcomes, and reported adverse events. Given the heterogeneity in study design and clinical endpoints, the synthesis followed a narrative and descriptive approach rather than a quantitative meta-analysis.

**Figure 1:**
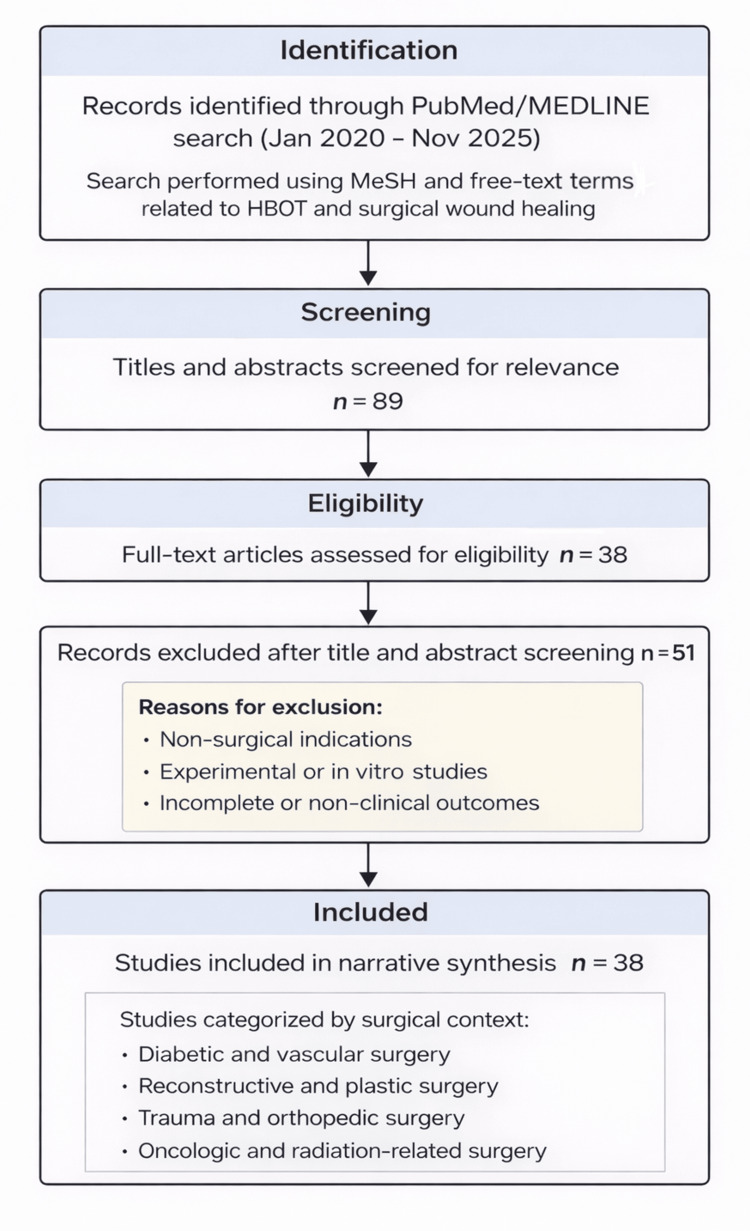
Literature identification, screening, eligibility assessment, and inclusion process for the narrative review of HBOT in surgical wound management MeSH: Medical Subject Headings; HBOT: hyperbaric oxygen therapy

Of the 89 studies identified, 51 were excluded after title and abstract screening because they did not meet the inclusion criteria (non-surgical indications, experimental models, or incomplete clinical outcomes). The remaining 38 studies constituted the complete set of included studies and references, and their identification, screening, and selection process is summarized in Figure 1.

Central Body

Physiological and Molecular Mechanisms of HBOT in Wound Healing

Cellular and biochemical responses to hyperoxia: HBOT enhances tissue repair through multiple, interrelated physiological and molecular pathways that collectively target the key barriers to post-surgical wound healing, namely, tissue hypoxia, inflammation, and infection. By exposing patients to 100% oxygen under pressures greater than one ATA, HBOT markedly increases the amount of oxygen dissolved in plasma, thereby raising tissue oxygen tension to levels that support reparative cellular processes.

This transient hyperoxia stimulates fibroblast proliferation, promotes collagen synthesis, accelerates keratinocyte migration, and facilitates overall re-epithelialization [4,8,9]. Experimental and clinical models consistently demonstrate that HBOT enhances granulation tissue formation, promotes extracellular matrix maturation, and reduces interstitial edema [4,9,10]. These effects are dose-dependent within the therapeutic range of 2.0-2.5 ATA for 60-90 minutes per session and are most pronounced in tissues with significant oxygen debt, such as ischemic flaps, poorly perfused graft beds, or irradiated fields [5,9]. By restoring the oxygen gradient required for hydroxylation reactions in collagen biosynthesis and for ATP-dependent cell migration, HBOT directly alleviates the biochemical bottlenecks that limit early postoperative repair in compromised tissues.

Modulation of inflammation and oxidative stress: Beyond increasing oxygen availability, HBOT exerts profound immunomodulatory and anti-inflammatory effects that facilitate wound progression from the inflammatory to the proliferative phase. Controlled hyperoxia induces a transient rise in reactive oxygen and nitrogen species that function as secondary messengers, activating transcription factors involved in cellular repair rather than causing oxidative damage [11,12]. This hormetic redox response results in the downregulation of pro-inflammatory cytokines such as TNF-α and IL-1β, suppression of NF-κB activation, and increased production of anti-inflammatory mediators that promote resolution [13,14]. 

HBOT also enhances macrophage polarization toward a reparative M2 phenotype and decreases adhesion molecule expression (ICAM-1, VCAM-1), thereby reducing leukocyte migration and tissue edema [14]. Clinical observations and animal studies have reported decreased expression of inflammatory biomarkers and improved tissue oxygen gradients after HBOT sessions, particularly in radiation-induced and ischemic wounds [12,15]. Additionally, HBOT modulates hypoxia-inducible factor-1α (HIF-1α) stability, preserving angiogenic signaling while avoiding chronic hypoxic stress that perpetuates inflammation [11,13]. Together, these mechanisms establish an anti-inflammatory and antioxidative environment conducive to effective wound healing.

Angiogenesis, neovascularization, and matrix remodeling: A central mechanism by which HBOT accelerates wound healing is the stimulation of angiogenesis and extracellular matrix remodeling. Under hyperoxic conditions, endothelial cells exhibit enhanced proliferation, migration, and tube formation, supported by the upregulation of vascular endothelial growth factor (VEGF) and endothelial nitric oxide synthase (eNOS) [16,17]. The transient rise in tissue oxygen tension promotes fibroblast activity and collagen cross-linking, improving the tensile strength and elasticity of the healing tissue [9,18]. HBOT also modulates the matrix metalloproteinase/tissue inhibitor of metalloproteinase (MMP/TIMP) balance, ensuring controlled extracellular matrix turnover and the orderly deposition of collagen types I and III, critical determinants of scar quality and stability [17,19]. 

Experimental and clinical studies across reconstructive and aesthetic surgery confirm that HBOT increases microvascular density and enhances flap and graft survival. In animal models and patient series, treated tissues demonstrate thicker collagen bundles, higher vessel density, and improved capillary maturity compared with non-treated controls [9,16-18]. These findings align with meta-analyses reporting superior outcomes in ischemic and postoperative wounds when HBOT is used as an adjuvant to conventional therapy [17,19]. 

Antimicrobial and immunomodulatory effects: In addition to promoting tissue regeneration, HBOT enhances the host immune response and exhibits direct and indirect antimicrobial effects. The hyperoxic environment augments the oxidative burst of neutrophils and macrophages, improving bacterial killing through myeloperoxidase activation and enhanced phagocytosis [13,20]. Elevated tissue oxygen tension also inhibits the growth of anaerobic microorganisms and disrupts bacterial biofilms, increasing susceptibility to antibiotic therapy [13,21]. These mechanisms contribute to reduced bioburden and decreased rates of surgical site infection, particularly in ischemic and contaminated wounds.

At the same time, HBOT modulates the inflammatory milieu by downregulating pro-inflammatory cytokines and adhesion molecules, thereby mitigating tissue edema and secondary necrosis. Clinical and experimental data have demonstrated significant reductions in bacterial load, enhanced local perfusion, and faster resolution of infection-related inflammation following adjunctive HBOT [20,21]. This dual antimicrobial and immunomodulatory action is clinically relevant in scenarios such as necrotizing soft tissue infections, post-radiation wounds, and ischemic ulcers, where oxygen tension is critically low and bacterial proliferation is favored [13,22,23]. Consequently, HBOT not only supports antibiotic efficacy but also restores the microenvironment required for host immune competence and effective tissue repair.

Integration with surgical physiology (timing, dose, and tissue context): The physiological benefits of HBOT are highly dependent on the timing of administration and the condition of the surgical tissue. Preconditioning with HBOT before reconstructive or complex re-operative procedures increases tissue oxygen reserve, enhances antioxidant defenses, and upregulates angiogenic mediators, improving tolerance to ischemia and surgical stress [24]. Early postoperative therapy, ideally within the first 48-72 hours, attenuates ischemia-reperfusion injury, decreases edema, and stabilizes microcirculatory flow, thereby preserving graft and flap viability [19,25,26]. These effects are particularly evident in lower-extremity trauma, crush injuries of the hand, and reconstructive procedures in hypoxic beds [19,27]. 

Standard clinical regimens typically employ pressures between 2.0 and 2.5 ATA for 60-90 minutes per session with air breaks, totaling 20-40 sessions depending on wound severity and tissue perfusion [26,28]. Adjusting the number and frequency of sessions according to ischemic burden and comorbidities improves efficacy and safety. Beyond monotherapy, HBOT demonstrates synergy when integrated with regenerative and mechanical modalities such as platelet-rich plasma (PRP), negative pressure wound therapy (NPWT), or extracorporeal shockwave therapy (ESWT). Evidence from comparative and randomized studies indicates that these combined strategies accelerate granulation, enhance collagen deposition, and shorten healing time compared with single-modality interventions [16,28,29]. 

Safety, mechanism-linked adverse events, and therapeutic window: HBOT is considered a safe therapeutic modality when applied within recommended pressure-time parameters. The majority of adverse events are mild, transient, and mechanistically linked to oxygen exposure or pressure variations rather than systemic toxicity. The most frequently reported effects include reversible myopia and middle-ear barotrauma, both of which resolve after therapy discontinuation or conservative management [5,7]. Oxygen-induced seizures are rare and self-limited, generally occurring only when pressures exceed 3.0 ATA or session duration surpasses standard protocols. Studies across surgical, reconstructive, and radiation-related settings confirm that adherence to treatment guidelines, 2.0-2.5 ATA for 60-90 minutes with air breaks, maintains an excellent safety profile [5,18,30]. 

From a physiological standpoint, hyperoxia exerts predictable vasoconstrictive effects that transiently reduce blood flow but do not impair microcirculatory perfusion; in fact, tissue oxygen delivery remains elevated because of the markedly increased plasma oxygen content [7,12]. In irradiated and ischemic tissues, these transient vascular changes are outweighed by enhanced oxygen diffusion, neovascularization, and bacterial clearance, leading to improved wound outcomes without evidence of long-term harm [18,30]. The overall benefit-risk ratio of HBOT is therefore most favorable in patients with documented tissue hypoxia, compromised flaps, or radiation injury, contexts in which the therapeutic window between efficacy and toxicity is wide and clinically manageable [5,7,12,18]. 

These mechanistic insights provide a physiological foundation for the clinical use of HBOT across surgical specialties, guiding evidence-based integration into perioperative protocols.

Clinical Evidence Across Surgical Specialties

Diabetic and vascular surgery (diabetic foot ulcer (DFU), chronic limb-threatening ischemia (CLTI), and venous/ischemic ulcers): Across diabetic and ischemic wound phenotypes, adjunctive HBOT consistently shows signals of improved healing trajectories and limb preservation when added to standard care. Multiple quantitative syntheses report benefits in healing and amputation-related outcomes in DFUs: a traditional meta-analysis of controlled trials demonstrated improved clinical endpoints with HBOT [30], an updated meta-analysis confirmed efficacy on key wound outcomes [1], and more recent evidence syntheses, both a broad systematic review of DFU modalities and a network meta-analysis, continue to position HBOT among the top adjuvant options depending on baseline risk and comparator [11,16]. In DFUs, a recent prospective comparative study reported faster epithelialization, more robust granulation, and fewer minor amputations when HBOT was added to standard wound care compared with standard care alone [7]. For diabetic foot patients requiring graft-based reconstruction, adjunctive HBOT has been associated with improved graft take and defect closure in cohort analyses, aligning mechanistically with enhanced angiogenesis and infection control in hypoxic beds [31-33].

In the spectrum of lower-limb ischemia and venous disease, the signal remains favorable. A systematic review of venous leg ulcers indicates potential benefit of HBOT as an adjunct while acknowledging heterogeneity in protocols and endpoints [31]. Real-world retrospective data over six years also suggest improved healing in venous leg ulcers treated with HBOT within single-center programs [23]. In resistant venous leg ulcers, comparative work shows that adding systemic HBOT to venous interventions can improve outcomes versus intervention alone, underscoring a role for oxygenation in recalcitrant disease [32]. 

Trial infrastructure is evolving to clarify where HBOT provides the greatest incremental value in ischemic limbs. The HOTFOOT multicenter randomized protocol integrates HBOT with endovascular therapy for CLTI, reflecting a contemporary shift toward hybrid limb-salvage pathways in which oxygen therapy is coordinated with revascularization and wound care [34]. Together, these data support a graded clinical approach: deploy HBOT as an adjuvant in DFU and venous/ischemic ulcers with documented hypoxia or stalled healing, and embed it within multidisciplinary limb-salvage algorithms that include revascularization, debridement, infection control, and off-loading [1,7,11,16,23,30-34]. 

Reconstructive and plastic surgery (flaps, grafts, burns, and aesthetic procedures): HBOT serves as a valuable adjunct in reconstructive and plastic surgery, particularly for compromised flaps, grafts, and ischemic or irradiated surgical beds. By increasing tissue oxygenation and stimulating angiogenesis, HBOT enhances flap viability, reduces ischemic necrosis, and improves wound closure. Clinical studies demonstrate that adjunctive HBOT enhances perfusion and endothelial proliferation, increases capillary density, and decreases the incidence of partial flap loss in complex reconstructive procedures [18,19]. These effects are especially relevant in nipple-sparing mastectomy reconstructions and similar flap-based surgeries, where HBOT contributes to improved survival of threatened flaps and reduced need for reoperation [18,22]. 

In burn management, HBOT provides both microvascular and anti-inflammatory benefits. Experimental and clinical studies reveal that HBOT decreases edema, reduces bacterial contamination, and improves epithelial regeneration in thermal injuries [14,20]. The modulation of inflammatory mediators such as ICAM-1 and enhanced neovascularization are key factors supporting accelerated re-epithelialization and graft integration. These findings are corroborated by histologic analyses showing greater collagen alignment and reduced necrosis in treated tissues [14,20].

The aesthetic and elective domains have also adopted HBOT to optimize postoperative outcomes and minimize complications. Evidence from systematic reviews and meta-analyses reports lower rates of flap congestion, bruising, and infection, together with improved scar quality and faster recovery [17,35]. Additionally, in combination with autologous regenerative techniques such as PRP, HBOT enhances fibroblast activation, matrix deposition, and angiogenic signaling, creating an oxygen-enriched environment that promotes tissue remodeling and aesthetic consistency [16,17]. Collectively, these findings confirm HBOT as a physiological enhancer of flap and graft survival while reducing complication rates across reconstructive, burn, and aesthetic surgery [9,14,16-20,22,35-37]. 

Trauma and orthopedic surgery: In traumatic and orthopedic settings, HBOT functions as an adjuvant that mitigates ischemia-reperfusion injury, improves microvascular perfusion, and enhances tissue salvage. In lower-extremity trauma, its early postoperative use has been linked to improved flap and graft viability, reduced infection, and shorter hospitalization. Clinical data show that adding HBOT to complex reconstructive protocols promotes capillary regeneration and accelerates healing in soft tissue defects [19]. 

In crush injuries of the hand and forearm, early HBOT reduces edema and pain, stabilizes tissue oxygenation, and improves functional recovery. A prospective analysis demonstrated faster resolution of necrosis and better range of motion among patients treated within the first 48 hours after injury [27]. Similar benefits have been reported in pediatric and rare hematologic cases, where HBOT supported the healing of surgical complications in hypoperfused tissue [38].

Experimental and case-series evidence in trauma-related reconstructive procedures also indicates that HBOT contributes to enhanced fibroblast proliferation, collagen alignment, and bone-tendon interface healing, favoring earlier mobilization and reduced need for revision surgeries [37]. Taken together, these findings reinforce the role of HBOT as an adjunct for trauma-related reconstructive surgery, particularly when tissue viability is threatened by crush injury, compartment syndrome, or compromised perfusion [19,27,36,38]. 

Oncologic and radiation-related surgery: HBOT has demonstrated significant therapeutic value as an adjunct in oncologic and radiation-related reconstructive surgery. Post-radiation wounds and soft tissue necrosis represent challenging scenarios due to chronic hypoxia, fibrosis, and impaired angiogenesis. HBOT counteracts these effects by increasing tissue oxygen tension, promoting neovascularization, and enhancing fibroblast function within irradiated beds. High-level evidence confirms its efficacy in the management of late radiation tissue injury, including osteoradionecrosis and soft tissue radionecrosis, by accelerating granulation tissue formation and improving wound closure rates [30].

In gynecologic oncology, HBOT is used as a supportive therapy for radiation-induced cystitis, proctitis, and pelvic tissue necrosis. Clinical data show symptomatic relief, reduced bleeding, and improved mucosal healing in patients receiving HBOT for post-radiation complications, underscoring its capacity to restore vascular and epithelial integrity [12]. Similarly, in breast reconstruction following mastectomy, HBOT has been reported to improve flap survival in irradiated or ischemic tissue, decreasing the incidence of wound dehiscence and necrosis [18,22].

The integration of HBOT into multidisciplinary oncologic care thus offers a physiological solution to radiation-induced hypoxia and delayed wound healing. By enhancing microcirculatory recovery and reversing fibrosis, HBOT supports tissue viability and reconstruction success, particularly in complex post-radiation fields where conventional interventions often fail [12,18,22,30].

Table 1 summarizes representative clinical studies supporting the use of HBOT across surgical wound contexts.

**Table 1:** Representative clinical studies supporting the use of HBOT in surgical wound management HBOT: hyperbaric oxygen therapy; DFU: diabetic foot ulcer

Reference	Study design	Surgical context	HBOT protocol (as reported)	Main findings	Clinical implications
Sharma et al., 2021 [1]	Systematic review and meta-analysis	DFUs	Adjunctive HBOT (protocols varied)	HBOT was associated with improved ulcer healing outcomes compared with standard care alone	Supports HBOT as an adjunct in DFU with impaired healing
Zhang et al., 2022 [2]	Updated systematic review and meta-analysis	DFUs	Adjunctive HBOT	Confirmed beneficial effects of HBOT on wound healing endpoints	Reinforces evidence for HBOT in DFU management
Kranke et al., 2015 [3]	Cochrane systematic review	Chronic wounds	Adjunctive HBOT	Evidence suggested potential benefit in selected chronic wounds, with heterogeneity across studies	Highlights the need for careful patient selection
Lin et al., 2023 [30]	Cochrane systematic review	Late radiation tissue injury	Adjunctive HBOT	HBOT improved healing and symptom control in radiation-related soft tissue injury	Supports HBOT in post-radiation surgical wounds
Myrthong et al., 2024 [6]	Prospective comparative study	DFUs	HBOT + standard wound care vs. standard care	Faster wound healing and fewer complications with adjunctive HBOT	Demonstrates the clinical benefit of HBOT in DFU
Lalieu et al., 2021 [23]	Retrospective cohort (six-year single center)	Venous leg ulcers	Adjunctive HBOT	Improved wound healing outcomes in chronic venous ulcers	Suggests a role for HBOT in refractory venous ulcers
Nasr et al., 2023 [18]	Clinical cohort study	Nipple-sparing mastectomy flaps	Adjunctive HBOT	Improved flap salvage and reduced ischemic complications	Supports HBOT in compromised reconstructive flaps
Chang et al., 2024 [27]	Clinical study	Hand crush injuries	Early adjunctive HBOT	Reduced edema and improved tissue recovery	Supports early HBOT use in traumatic ischemic injuries

Analytical Summary and Critical Perspective

Despite the growing body of evidence supporting HBOT as a perioperative adjunct, interpretation of clinical outcomes remains limited by substantial heterogeneity in study design, dosing protocols, and patient selection criteria. Variations in treatment pressure (ranging from 2.0 to 3.0 ATA), session duration (60-120 minutes), and total exposure (10-60 sessions) complicate direct comparison and hinder meta-analytic consolidation. Moreover, the inclusion of diverse wound etiologies, namely, diabetic, ischemic, post-radiation, and traumatic, often under distinct pathophysiological contexts, introduces further variability. Standardization of HBOT parameters, coupled with consistent use of objective endpoints such as transcutaneous oxygen pressure (TcPO₂), angiogenic biomarkers, or validated wound healing scores, is essential for the next generation of clinical studies.

Future research should prioritize multicenter randomized designs and mechanistic translational studies that integrate clinical outcomes with molecular profiling of oxygen-responsive pathways. This integrative approach will not only refine patient selection and optimize dosing strategies but also clarify the mechanistic thresholds at which HBOT confers maximal benefit across surgical disciplines. Until such standardization is achieved, HBOT should be applied within structured, multidisciplinary frameworks, where physiological monitoring and individualized dosing guide its clinical implementation.

Limitations and Future Directions

This review has several limitations inherent to its narrative design. The synthesis relied exclusively on publications indexed in PubMed/MEDLINE, which, although comprehensive, may not capture all relevant data contained in other databases or grey literature. Nonetheless, PubMed remains the most authoritative source for peer-reviewed biomedical evidence, particularly in the surgical and hyperbaric medicine domains. The decision to limit the search to the past five years (2020-2025) was deliberate, ensuring the inclusion of contemporary studies using standardized HBOT protocols, but it also excludes earlier foundational work that contributed to the conceptual understanding of oxygen physiology and tissue repair. Therefore, while this time restriction enhances clinical relevance, it may limit historical depth.

Heterogeneity across included studies represents a second major constraint. Variations in HBOT parameters, such as treatment pressure, duration, frequency, and timing relative to surgery, impede direct comparison and quantitative synthesis. Moreover, differences in wound etiology, patient comorbidities, and outcome measures contribute to substantial methodological variability. Although this review sought to address these inconsistencies by emphasizing mechanistic and thematic synthesis, the absence of a uniform protocol across studies underscores the urgent need for standardized clinical guidelines. Establishing consistent dosing regimens and treatment algorithms would allow for more precise evaluation of HBOT efficacy across surgical contexts.

Another limitation concerns the predominance of small, single-center studies and retrospective designs within the current literature. Few randomized controlled trials have adequately powered patient populations to assess definitive clinical endpoints such as amputation rates, flap viability, or long-term functional recovery. To advance the field, multicenter prospective trials integrating molecular biomarkers of oxygen response, such as VEGF, HIF-1α, or inflammatory cytokine profiles, should be prioritized. Linking mechanistic outcomes with clinical metrics will enable a more nuanced understanding of which patients derive the greatest benefit from HBOT and how perioperative timing, tissue oxygenation status, and adjunctive therapies influence results.

Finally, future research should explore cost-effectiveness and protocol integration within perioperative and reconstructive care pathways. Given the logistical demands and resource intensity of HBOT, identifying optimal patient selection criteria and economically sustainable regimens is critical. A standardized, evidence-based framework, supported by translational science and validated clinical endpoints, will transform HBOT from an adjuvant option to an integral component of comprehensive surgical wound management.

Despite a growing body of convincing evidence supporting the clinical benefits of HBOT, its adoption into routine surgical practice remains limited. Several non-clinical factors contribute to this discrepancy. These include restricted availability of hyperbaric facilities, high infrastructural and operational costs, the need for specialized personnel, and limited integration of HBOT into standardized surgical pathways. Additionally, variability in reimbursement policies, lack of familiarity among surgeons and perioperative teams, and persistent heterogeneity in clinical protocols hinder broader implementation. Importantly, much of the current evidence is derived from single-center studies and heterogeneous trial designs, which may reduce confidence among policymakers and guideline committees. Addressing these barriers through standardized protocols, health economic analyses, and inclusion of HBOT within multidisciplinary care algorithms will be essential to translate existing evidence into widespread clinical adoption.

## Conclusions

HBOT is a scientifically grounded and clinically versatile adjunct for optimizing surgical wound healing. By modulating oxygen delivery, inflammation, angiogenesis, and host defense, HBOT directly addresses key physiological barriers to recovery in ischemic, infected, and irradiated tissues. Evidence across diabetic, reconstructive, traumatic, and oncologic surgery consistently supports its association with improved flap and graft viability, reduced infection rates, and enhanced wound closure.

Despite heterogeneity in study design and treatment protocols, the mechanistic rationale for HBOT remains compelling. By restoring tissue oxygen gradients essential for collagen synthesis, cellular proliferation, and microvascular repair, HBOT creates a pro-regenerative surgical microenvironment. Future progress will depend on standardized protocols, validated outcome measures, and biomarker-guided patient selection, allowing HBOT to evolve from an adjunctive option to an integral component of multidisciplinary surgical care.
